# Cold Snapshot of a Molecular Rotary Motor Captured by High‐Resolution Rotational Spectroscopy

**DOI:** 10.1002/anie.201704221

**Published:** 2017-06-20

**Authors:** Sérgio R. Domingos, Arjen Cnossen, Wybren J. Buma, Wesley R. Browne, Ben L. Feringa, Melanie Schnell

**Affiliations:** ^1^ Max Planck Institute for the Structure and Dynamics of Matter Luruper Chaussee 149 22761 Hamburg Germany; ^2^ Deutsches Elektronen-Synchrotron DESY Notkestrasse 85 22607 Hamburg Germany; ^3^ Stratingh Institute for Chemistry and Zernike Institute for Advanced Materials University of Groningen Nijenborgh 4 9747 AG Groningen The Netherlands; ^4^ Van't Hoff Institute for Molecular Sciences University of Amsterdam Science Park 904 1098 XH Amsterdam The Netherlands; ^5^ Christian-Albrechts-Universität zu Kiel Institute of Physical Chemistry Max-Eyth-Strasse 1 24118 Kiel Germany

**Keywords:** high-resolution spectroscopy, large molecules, microwave spectroscopy, molecular motors, structure elucidation

## Abstract

We present the first high‐resolution rotational spectrum of an artificial molecular rotary motor. By combining chirped‐pulse Fourier transform microwave spectroscopy and supersonic expansions, we captured the vibronic ground‐state conformation of a second‐generation motor based on chiral, overcrowded alkenes. The rotational constants were accurately determined by fitting more than 200 rotational transitions in the 2–4 GHz frequency range. Evidence for dissociation products allowed for the unambiguous identification and characterization of the isolated motor components. Experiment and complementary quantum‐chemical calculations provide accurate geometrical parameters for the C_27_H_20_ molecular motor, the largest molecule investigated by high‐resolution microwave spectroscopy to date.

Inspired by Nature's ability to perform motor functions at the molecular level, chemists have engaged in the design of synthetic nanomachines that can perform molecular motion in a controlled manner and mimic their biological counterparts using simpler models.^1^, ^2^ An elegant design of a synthetic rotary molecular motor based on chiral, overcrowded alkenes was introduced by Feringa and co‐workers.^3^ Key features of this design include 1) a light‐activated power stroke in which excited‐state *cis*–*trans* isomerization converts photon energy into mechanical motion and 2) a chiral center that imposes unidirectional motion departing from conventional molecular photoswitching. The operation mechanism of such a motor is illustrated in Figure 1. The system is comprised of a “stator” fluorene unit connected to an upper “rotor” via a C=C “axle”. An ultraviolet trigger results in photoisomerization of the axle, leading to a rotation of the rotor with respect to the stator. This motion yields isomer **1‐B**. The methyl group at the chiral center now adopts a pseudoequatorial conformation while that of **1‐A** is pseudoaxial. A thermally activated helix inversion returns the methyl group to the more energetically favorable pseudoaxial orientation, **1‐C**. This step reintroduces the steric hindrance, locks the rotor, and ensures unidirectional rotation in the forward direction.

**Figure 1:**
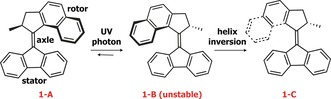
Structure of the molecular motor. The three components, namely rotor, axle, and stator, are indicated as well as the light‐driven (power) and thermal strokes required for operation. Further photon‐driven and thermal isomerization events return the motor to its original configuration **1‐A**.

The synthesis of nanomachines, such as the one investigated here, marks an era where small artificial molecular constructs are able to perform mechanical work. Rotaxane‐based systems^4^, ^5^ and unidirectional rotary molecular motors^6^, ^7^ are among the systems designed to perform translational and rotary motion, respectively. The functional performance of these nanomachines clearly emerges from their unique structural properties. Further understanding and optimizing such molecular machinery are therefore largely dependent on the ability to get detailed information on the molecular conformations of the key mechanical steps and their structural evolution, preferably under conditions where the system is not perturbed by external influences. Experimental techniques that have thus far been employed for the structure elucidation of such molecular machines include NMR,^8^ time‐resolved IR,^9^ fluorescence, and electronic^10^ spectroscopies. They provide important information but do not meet up to the last requirement. High‐resolution rotational spectroscopy on isolated nanomachines in the gas phase, on the other hand, is preeminently suited for this purpose but the largest molecular systems that have been studied with these techniques hardly come close to the molecular motor that is considered here with respect to the number of non‐hydrogen atoms.^11^, ^12^, ^13^

Herein, we report on the first high‐resolution rotational spectrum of a molecular motor, which was obtained by combining microwave spectroscopy with the cold conditions of a supersonic jet. Microwave spectroscopy enables the unambiguous identification of molecular species and the determination of the thermal distribution of conformations. Owing to their unique moments of inertia, each conformation of a particular molecule can be differentiated by its rotational spectrum. With the implementation of short and intense microwave chirps in broadband excitation schemes as in chirped‐pulse Fourier transform microwave (CP‐FTMW) spectroscopy, it is possible to record rotational spectra of complex, flexible molecules spanning several GHz in a single acquisition.^14^

The cold molecular jet brings the molecules to rotational temperatures below 2 K, which for a molecular system of this size (C_27_H_20_, *M*_w_=344 g mol^−1^) implies that the strongest rotational transitions are situated between 2 and 4 GHz. The broadband microwave spectrum of **1‐A** in this region is shown in Figure 2. The experimental spectrum is shown as the upper trace (in black). The spectrum shown below (in red) represents a simulation obtained from the fitted spectroscopic parameters reported in Table 1. The right panel of this Figure displays a segment of the rotational spectrum, highlighting a branch of rotational transitions $J_{K_{a}K_{c}}\leftarrow J{{}^{'}}_{K_{a{}^{'}}K_{c{}^{'}}}$ denoted by the rotational quantum numbers *J*, *K_a_*, and *K_c_*, with *J* being the rotational angular momentum quantum number and *K_a_* and *K_c_* being the projections of *J* onto the principal axes at the prolate and oblate symmetric top limits, respectively. A total of 222 rotational transitions were assigned, and the primary rotational constants (*A*, *B*, *C*) were determined through a recurrent fit using the A‐reduced semirigid rotor Hamiltonian as implemented in PGOPHER.^15^ Quartic centrifugal distortion constants (*D_J_* and *d_J_*) were also determined. We note that the inclusion of distortion constants is not required to achieve a good fit. The small magnitudes obtained for both *D_J_* and *d_J_* are a strong indicator of the rigidity of the molecule in spite of its size. A summary of the fitted spectroscopic parameters is given in Table 1 while a complete list of all fitted rotational transitions is provided in the Supporting Information. We found neither evidence for internal dynamics arising from the methyl top nor for other large‐amplitude motions, which would point to high barriers associated with these motions. As the methyl group is part of the ratchet during the operation of the motor, this is indeed what would be expected.

**Figure 2:**
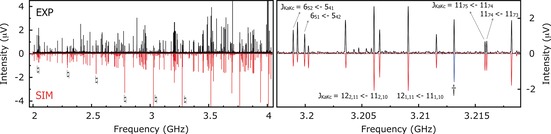
Broadband rotational spectrum of **1**‐**A** from 2 to 4 GHz (1.5 million averages, measurement time: 13 h). The upper trace (in black) shows the experimental spectrum obtained using neon as the carrier gas. The lower trace represents simulations obtained from the fitted spectroscopic parameters reported in Table 1. The 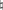 marks the a/b quartet progressions that are shown in detail in Figure 3. The rotational transition marked with † corresponds to the dissociation structure of the rotor as a consequence of fragmentation (see main text). The spectroscopic parameters of the fragment are reported in Table 2.

**Table 1:** Experimentally determined parameters for the vibronic ground state of the motor identified in the microwave spectrum.^[a]^

	Exp.	M06‐2X^[b]^	MP2^[b]^	B3LYP^[b]^	B3LYP‐D3BJ^[c]^
*A* [MHz]	307.183437(46)	308.806	305.187	306.565	308.633
*B* [MHz]	164.951398(47)	165.639	168.546	162.795	166.282
*C* [MHz]	122.506084(33)	122.462	124.127	121.704	122.875
*D_J_* [kHz]	0.001431(90)	–	–	–	–
*d_J_* [kHz]	0.000271(50)	–	–	–	–
|*μ_a_*| [D]	y	1.28	1.31	1.39	1.37
|*μ_b_*| [D]	y	0.99	0.55	1.03	0.99
|*μ_c_*| [D]	n	0.12	0.05	0.15	0.11
*N*	222	–	–	–	–
*σ* [kHz]	3.4	–	–	–	–
*κ*	−0.540	−0.536	−0.509	−0.555	−0.532

[a] Rotational constants (*A*, *B*, *C* in MHz) and quartic centrifugal distortion constants (in kHz); type of spectrum observed (a‐type, b‐type, c‐type) with y being observed and n being not observed; predicted dipole moments; number of lines used in the fit; standard error of the fit (in kHz); asymmetry parameter *κ*=(2B‐A‐C)/(A‐C). The experimental frequency accuracy is 25 kHz. [b] 6‐311++G** basis set. [c] def2‐TZVP basis set.

In our frequency range, we cover mainly a‐ and b‐type transitions. In Figure 3, we show segments of the spectrum depicting a progression of a/b‐type quartets over the range (*J*+1)←*J* of 8←7 to 13←12. Following the progression from lower to higher frequencies (panels A→F), we observe a narrowing between transitions, resulting in coalescence for 13←12 further up the *J* levels.

**Figure 3:**
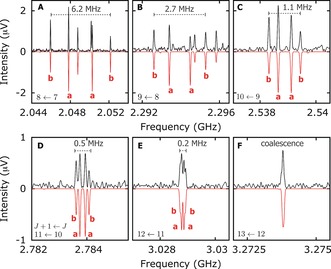
Segments A→F of the rotational spectrum following the frequency regions marked with in Figure 2. The characteristic a/b quartets are nicely resolved for *J*+1←*J=*8←7 (A) and reach complete coalescence for *J*+1←*J=*13←12 (F).

In Table 1, we compare the results taken from our observations with a series of quantum‐chemical calculations at different levels of theory (see the Supporting Information for further details). We found a very good agreement between theoretical predictions and our experimental observations at all levels of theory. The most impressive match between the experimental and calculated rotational constants was obtained at the M06‐2X/6‐311++G** level of theory, for which experiment and theory differ by less than 0.5 % for all three rotational constants. In addition, the magnitudes of the permanent dipole moment components are in good agreement with the observed intensities. At the same time, the dispersion‐corrected B3LYP‐D3BJ level of theory predicts the experimental rotational constants equally well, in particular if one considers the deviations (ca. 1 %) predicted for vibrationally corrected rotational constants with respect to the equilibrium ones.^16^

The excellent agreement between experiment and theory enabled us to determine key geometrical parameters of the molecular motor. The length of the C=C bond of the motor is 1.356 Å (M06‐2X), which is very similar to the length determined from the crystal structure^6^ (1.357 Å). Comparison of this bond length with those of other non‐sterically overcrowded alkenes, such as ethylene (C_2_H_4_, 1.325 Å) and 2‐butene (C_4_H_8_, 1.329 Å), readily indicates that the C=C link is substantially extended in the molecular motor. To evaluate the local geometry around the axle, we defined three planes (Figure 4), which comprise the planar part of the stator (in yellow), the axle and rotor (in blue), and the planar part of the rotor (in red), respectively. The angles *α*=50.8° (50.0°), *β*=42.0° (39.6°), and *γ*=18.4° (22.8°) define the relative twisting of the rotor with respect to the stator in the locked conformation (crystal structure values are given in parentheses). Comparison with the values obtained from the crystal structure shows that the structure of the motor is unmistakably affected by its environment. The gas‐phase dihedral angle at the axle coordinate differs by approximately 1.6° from the crystal structure, with *D*(2‐3‐4‐5)=13.5° (15.18°).

**Figure 4:**
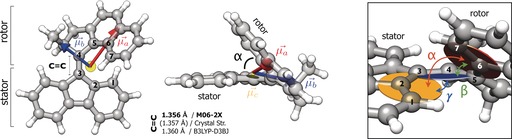
Molecular structure of the rotor (**1‐A**) obtained at the M06‐2X/6‐311++G** level of theory. Relevant geometrical parameters are given; the C=C bond length is 1.356 Å (the value in parentheses refers to the length taken from the crystal structure),^6^ the depicted planes with respect to the respective numbered atoms (yellow: 1‐2‐3; blue: 3‐4‐5; red: 5‐6‐7) define the local torsions around the axle. The angles between the planes are *α*=50.8°, *β*=42.0°, and *γ*=18.4°. (*α*,*β*,*γ*)_B3LYP‐D3BJ_=(*α*,*β*,*γ*)_M06‐2X_+0.2°. The dihedral angle *D*(2‐3‐4‐5) is 13.5°(M06‐2X)/13.8°(B3LYP‐D3BJ).

Interestingly, we also found evidence that under our experimental conditions, some fragmentation of the molecular motor occurs. In the rotational spectrum, we identified and fitted a series of rotational lines that correspond to dissociation products of the rotor and the stator. Fragmentation occurs owing to preexpansion heating at the nozzle. One of the 23 rotational transitions belonging to the rotor moiety is shown in Figure 2 (right panel). The spectroscopic parameters obtained from the fit to these transitions are given in Table 2. Quantum‐chemical calculations on two tentative models for a dissociation product of the rotor confirmed our expectations: from the comparison of rotational constants, dipole moment components, and asymmetry parameters, we unambiguously identified **r‐B** as the structure of the fragment. Dissociation thus results in a planar rotor fragment that no longer possesses a chiral center as compared with the geometry of the rotor when it is coupled to the motor. The stator fragment was unambiguously assigned to fluorene based on a fit using 18 rotational transitions and a direct comparison with reported rotational constants.^17^ The spectroscopic parameters are reported in the Supporting Information.

**Table 2:** Experimental and calculated spectroscopic parameters of the rotor fragment.^[a]^

	Exp.	r‐A^[b]^	r‐B^[b]^
*A* [MHz]	1591.057(12)	1469.574	1598.998
*B* [MHz]	488.12288(21)	515.633	489.503
*C* [MHz]	375.37307(20)	408.807	376.520
|*μ_a_*| [D]	y	0.69	1.45
|*μ_b_*| [D]	n	0.17	0.01
|*μ_c_*| [D]	n	0.16	0.00
*N*	23	–	–
*σ* [kHz]	3.9	–	–
*κ*	−0.81	−0.79	−0.81
		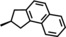	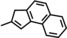

[a] Rotational constants (*A*, *B*, *C* in MHz); type of spectrum observed (a‐type, b‐type, c‐type) with y being observed and n being not observed; predicted dipole moments; number of lines used in the fit; standard error of the fit (in kHz); asymmetry parameter *κ*=(2B‐A‐C)/(A‐C). The experimental frequency accuracy is 25 kHz. [b] M06‐2X/6‐311++G**.

In summary, we have presented the first high‐resolution rotational spectrum of a molecular rotary motor and used it to determine the exact conformation of the motor in its vibronic ground state and to derive key structural parameters. Rotational constants were determined with high accuracy and provide an excellent basis for benchmarking the current levels of theory implemented in quantum‐chemical methods for large molecular systems. Temperature‐induced fragmentation of the motor has allowed us to observe the motor components separately and analyze the structures of the dissociation products. The unprecedented observation of a molecule of this size by microwave spectroscopy introduces exciting perspectives to the investigation of other molecules of similar and larger sizes by high‐resolution spectroscopy. In the present study, we have reported on the conformation of the motor in the absence of an external trigger and observed a single conformer corresponding to the thermally stable ground‐state structure. These studies served to demonstrate the feasibility of high‐resolution rotational spectroscopic studies on systems of this size. Experiments that have now come within reach use UV photons to drive the initial power stroke of the motor and combine this activation of the motor with the high resolving power of rotational spectroscopy to determine the structure of intermediate metastable mechanical steps in the photocycle of these molecular machines. Such experiments are presently being set up in our laboratories.

## Conflict of interest

The authors declare no conflict of interest.

## Supporting information

As a service to our authors and readers, this journal provides supporting information supplied by the authors. Such materials are peer reviewed and may be re‐organized for online delivery, but are not copy‐edited or typeset. Technical support issues arising from supporting information (other than missing files) should be addressed to the authors.

Supplementary
