# Supplementary material for: Cold Snapshot of a Molecular Rotary Motor Captured by High‐Resolution Rotational Spectroscopy
Source: Angew Chem Int Ed Engl. 2017 Jun 20;56(37):11209–12. doi: 10.1002/anie.201704221 (PMC5599986; doi:10.1002/anie.201704221)
Supplement: Supplementary file 1 — Supplementary [file ANIE-56-11209-s001.pdf]

Angewandte Chemie
Eine Zeitschrift der Gesellschaft Deutscher Chemiker

Supporting Information

Cold Snapshot of a Molecular Rotary Motor Captured by High-Resolution Rotational Spectroscopy

*Sérgio R. Domingos, Arjen Cnossen, Wybren J. Buma, Wesley R. Browne, Ben L. Feringa, and Melanie Schnell**

anie_201704221_sm_miscellaneous_information.pdf

Contents

1 Experimental details **2**

2 Theoretical details **2**

3 Rotational spectra of the dissociation products **3**

4 Line lists **3**

4.1 Molecular Motor 3

4.2 Rotor fragment (r-B) 10

1 Experimental details

We use broadband rotational spectroscopy to investigate the rotational spectrum of molecular motor 1-A using chirped-pulse Fourier transform microwave spectroscopy (CP-FTMW). The spectrum was recorded using the Hamburg COMPACT spectrometer as described elsewhere.[1] The sample was synthesised and purified as previously described.[2] A supersonic expansion brings the sample into the vacuum chamber where a 4 μs chirp spanning 2−4 GHz polarises the ensemble of molecules. The seeding of molecules into a supersonic expansion is done using a pulsed nozzle (Parker General Valve Series 9) operating at 3 Hz with a constant flow of Ne at stagnation pressures of 0.5 bar to generate a cold molecular jet. To create sufficient vapour pressure the sample was heated to 180°C directly at the nozzle. The chirp is generated with an arbitrary waveform generator and amplified in a 300 W traveling wave tube amplifier (2.5−7.5 GHz) before being broadcast into the vacuum chamber using a horn antenna. For this particular molecular system, a shorter chirp was produced in the arbitrary waveform generator covering only 2 GHz instead of the 6 GHz normally utilised in our spectrometer. In this way we effectively allocate the entire 300 W amplification power over the frequency region of interest and improve our signal-to-noise ratio. Upon chirped microwave excitation we record the free induction decay (FID) of the macroscopic dipole moment of the ensemble of molecules. In our experiments we used a new data acquisition scheme using the 'fast frame' option of the digital oscilloscope.[3] In short, eight back-to-back excitation chirps are performed on each sample pulse, and the subsequent eight FID acquisitions are co-added and averaged. This scheme decreases the measurement time and sample consumption, resulting in an effective repetition rate of 24 Hz for this particular experiment. We record 40 μs of the FIDs which, after a Fourier transformation (FT), gives a resolution of 25 kHz in the microwave spectrum. 1.5 M FIDs (equivalent to 13 hours of measurement time) were co-added to obtain the final spectrum.

2 Theoretical details

Geometry optimisations were performed using second-order Møller-Plesset perturbation theory (MP2), and Density Functional Theory using the hybrids B3LYP (Becke, three-parameter, LeeYangParr), B3LYP-D3BJ (including Grimme's dispersion correction[4] with Becke-Johnson damping[5]) and the M06-2X exchange-correlation energy functional[6]. Basis sets 6-311++G** and def2-TZVP were used. All calculations were done using Gaussian09.[7]

3 Rotational spectra of the dissociation products

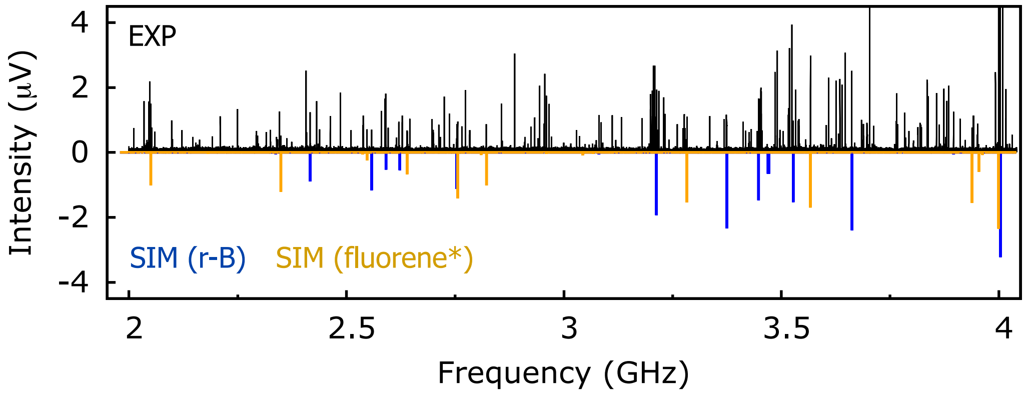

Figure 1: Broadband rotational spectrum of the molecular motor from 2 to 4 GHz (1.5M averages, 13 h of measurement time). The upper trace (in black) shows the experimental spectrum using neon as carrier gas. The lower trace represents simulations obtained from the fitted spectroscopic parameters of the dissociation structure of the rotor (r-B, see Table 2 in main paper). [*] The simulated spectrum of fluorene was obtained directly using the rotational constants reported in Ref 16 (A=2176.210160(70) MHz, B=586.65338(12) MHz, C=463.568993(63) MHz).

4 Line lists

4.1 Molecular Motor

Table 1: Observed and calculated rotational transitions (MHz) for the molecular motor. A few additional strong transitions above 4 GHz were included in the fit and those are listed at the end of the table.

Observed	Calculated	Obs-Calc	J′	K_a′	K_c′	J″	K_a″	K_c″
2010.8533	2010.8533	0.0000	7	1	6	6	1	5
2034.4841	2034.4839	0.0002	7	3	5	6	3	4
2041.5154	2041.5134	0.0020	7	5	3	6	5	2
2042.0136	2042.0112	0.0024	7	5	2	6	5	1
2045.9386	2045.9391	-0.0005	8	0	8	7	1	7
2047.8200	2047.8219	-0.0019	8	1	8	7	1	7
2048.7712	2048.7728	-0.0016	7	4	4	6	4	3
2050.2737	2050.2747	-0.0010	8	0	8	7	0	7
2052.1574	2052.1574	0.0000	8	1	8	7	0	7
2059.8611	2059.8607	0.0004	7	4	3	6	4	2
2098.3786	2098.3792	-0.0006	7	2	6	6	1	5

2121.8058	2121.8018	0.0040	7	3	4	6	3	3
2146.6309	2146.6258	0.0051	7	2	5	6	2	4
2162.1850	2162.1868	-0.0018	8	1	7	7	2	6
2191.5020	2191.5063	-0.0043	5	3	3	4	2	2
2210.0580	2210.0589	-0.0009	8	2	7	7	2	6
2249.7094	2249.7127	-0.0033	8	1	7	7	1	6
2292.6050	2292.6069	-0.0019	9	0	9	8	1	8
2293.4025	2293.4032	-0.0007	9	1	9	8	1	8
2294.4840	2294.4896	-0.0056	9	0	9	8	0	8
2294.7196	2294.7245	-0.0049	4	4	1	3	3	0
2295.2846	2295.2860	-0.0014	9	1	9	8	0	8
2295.8332	2295.8302	0.0030	4	4	0	3	3	1
2297.5851	2297.5848	0.0003	8	2	7	7	1	6
2315.8993	2315.8926	0.0067	8	3	6	7	3	5
2324.8689	2324.8700	-0.0011	5	3	2	4	2	3
2325.4931	2325.4936	-0.0005	8	7	1	7	7	0
2325.4931	2325.4927	0.0004	8	7	2	7	7	1
2339.6409	2339.6399	0.0010	8	5	4	7	5	3
2341.5602	2341.5680	-0.0078	8	5	3	7	5	2
2362.9619	2362.9638	-0.0019	9	2	7	8	3	6
2374.0197	2374.0187	0.0010	8	4	4	7	4	3
2407.0818	2407.0803	0.0015	6	3	4	5	2	3
2422.3076	2422.3177	-0.0101	9	8	2	9	7	3
2422.3076	2422.3100	-0.0024	9	8	1	9	7	2
2427.3536	2427.3507	0.0029	8	8	0	8	7	1
2427.3536	2427.3516	0.0020	8	8	1	8	7	2
2431.4156	2431.4172	-0.0016	8	2	6	7	2	5
2438.6315	2438.6283	0.0032	9	1	8	8	2	7
2463.0301	2463.0293	0.0008	9	2	8	8	2	7
2486.4999	2486.5004	-0.0005	9	1	8	8	1	7
2510.9013	2510.9014	-0.0001	9	2	8	8	1	7
2538.3198	2538.3205	-0.0007	10	0	10	9	1	9
2538.6513	2538.6505	0.0008	10	1	10	9	1	9
2539.1173	2539.1168	0.0005	10	0	10	9	0	9
2539.4430	2539.4469	-0.0039	10	1	10	9	0	9
2580.6479	2580.6447	0.0032	5	4	2	4	3	1

2588.4230	2588.4221	0.0009	5	4	1	4	3	2
2590.5655	2590.5682	-0.0027	9	3	7	8	3	6
2596.1191	2596.1204	-0.0013	7	3	5	6	2	4
2615.5834	2615.5828	0.0006	9	8	1	8	8	0
2615.5834	2615.5827	0.0007	9	8	2	8	8	1
2620.6211	2620.6235	-0.0024	9	7	2	8	7	1
2628.4458	2628.4432	0.0026	9	6	4	8	6	3
2628.7211	2628.7221	-0.0010	9	6	3	8	6	2
2639.6336	2639.6302	0.0034	9	5	5	8	5	4
2641.3991	2641.3975	0.0016	9	4	6	8	4	5
2645.5994	2645.5981	0.0013	9	5	4	8	5	3
2696.9377	2696.9338	0.0039	9	2	7	8	2	6
2699.5391	2699.5333	0.0058	6	3	3	5	2	4
2699.8432	2699.8376	0.0056	9	4	5	8	4	4
2712.5939	2712.5935	0.0004	10	2	9	9	2	8
2716.4993	2716.5050	-0.0057	10	2	8	9	3	7
2725.1810	2725.1838	-0.0028	10	1	9	9	1	8
2765.3868	2765.3872	-0.0004	8	3	6	7	2	5
2773.5023	2773.5026	-0.0003	9	3	6	8	3	5
2783.6127	2783.6125	0.0002	11	0	11	10	1	10
2783.7476	2783.7471	0.0005	11	1	11	10	1	10
2783.9447	2783.9425	0.0022	11	0	11	10	0	10
2784.0757	2784.0771	-0.0014	11	1	11	10	0	10
2856.2139	2856.2097	0.0042	6	4	3	5	3	2
2858.1561	2858.1656	-0.0095	10	3	8	9	3	7
2886.8990	2886.8988	0.0002	6	4	2	5	3	3
2910.3906	2910.3976	-0.0070	10	8	2	9	8	1
2924.5415	2924.5382	0.0033	9	3	7	8	2	6
2932.7219	2932.7223	-0.0004	10	4	7	9	4	6
2940.7568	2940.7582	-0.0014	10	5	6	9	5	5
2944.1127	2944.1094	0.0033	10	2	8	9	2	7
2954.4613	2954.4647	-0.0034	11	1	10	10	2	9
2956.2977	2956.3001	-0.0024	10	5	5	9	5	4
2959.9673	2959.9656	0.0017	11	2	10	10	2	9
2966.2780	2966.2753	0.0027	11	1	10	10	1	9
2971.7762	2971.7763	-0.0001	11	2	10	10	1	9

3028.7250	3028.7261	-0.0011	12	0	12	11	1	11
3028.7790	3028.7802	-0.0012	12	1	12	11	1	11
3028.8590	3028.8606	-0.0016	12	0	12	11	0	11
3028.9180	3028.9148	0.0032	12	1	12	11	0	11
3035.6481	3035.6466	0.0015	10	4	6	9	4	5
3048.9395	3048.9435	-0.0040	14	10	5	14	9	6
3065.6553	3065.6552	0.0001	12	10	3	12	9	4
3071.4315	3071.4398	-0.0083	11	10	2	11	9	3
3080.5058	3080.5058	0.0000	10	3	7	9	3	6
3085.7682	3085.7701	-0.0019	10	3	8	9	2	7
3111.1175	3111.1176	-0.0001	7	4	4	6	3	3
3119.0939	3119.1068	-0.0129	11	3	9	10	3	8
3132.4172	3132.4145	0.0027	7	3	4	6	2	5
3179.6201	3179.6175	0.0026	11	2	9	10	2	8
3181.1443	3181.1376	0.0067	12	3	9	11	4	8
3198.9441	3198.9450	-0.0009	6	5	2	5	4	1
3199.3219	3199.3209	0.0010	7	4	3	6	3	4
3199.9536	3199.9513	0.0023	6	5	1	5	4	2
3200.2848	3200.2875	-0.0027	11	9	2	10	9	1
3206.0685	3206.0694	-0.0009	12	2	11	11	2	10
3206.5672	3206.5680	-0.0008	11	8	3	10	8	2
3209.0817	3209.0831	-0.0014	12	1	11	11	1	10
3211.5694	3211.5703	-0.0009	12	2	11	11	1	10
3215.8593	3215.8588	0.0005	11	7	5	10	7	4
3216.0063	3216.0066	-0.0003	11	7	4	10	7	3
3218.1994	3218.1974	0.0020	11	4	8	10	4	7
3229.5456	3229.5460	-0.0004	11	6	6	10	6	5
3232.6365	3232.6352	0.0013	11	6	5	10	6	4
3241.7063	3241.6945	0.0118	11	5	7	10	5	6
3260.7682	3260.7675	0.0007	11	3	9	10	2	8
3276.5994	3276.6034	-0.0040	11	5	6	10	5	5
3330.9685	3330.9722	-0.0037	12	2	10	11	3	9
3335.3723	3335.3717	0.0006	8	4	5	7	3	4
3368.4168	3368.4174	-0.0006	11	3	8	10	3	7
3373.5335	3373.5342	-0.0007	11	4	7	10	4	6
3374.4343	3374.4361	-0.0018	12	3	10	11	3	9

3412.1272	3412.1222	0.0050	12	2	10	11	2	9
3450.4090	3450.4089	0.0001	13	1	12	12	2	11
3451.5058	3451.5072	-0.0014	13	2	12	12	2	11
3452.8961	3452.8961	0.0000	13	1	12	12	1	11
3453.9946	3453.9944	0.0002	13	2	12	12	1	11
3455.5885	3455.5861	0.0024	12	3	10	11	2	9
3485.5194	3485.5196	-0.0002	7	5	3	6	4	2
3490.2550	3490.2522	0.0028	12	10	2	11	10	1
3490.5311	3490.5283	0.0028	7	5	2	6	4	3
3496.0387	3496.0367	0.0020	12	9	4	11	9	3
3496.0387	3496.0372	0.0015	12	9	3	11	9	2
3496.5784	3496.5698	0.0086	12	4	9	11	4	8
3516.3502	3516.3507	-0.0005	12	7	6	11	7	5
3516.8575	3516.8648	-0.0073	12	7	5	11	7	4
3518.7931	3518.8025	-0.0094	14	0	14	13	0	13
3524.0946	3524.0989	-0.0043	6	6	0	5	5	1
3525.1343	3525.1351	-0.0008	9	4	6	8	3	5
3532.9037	3532.9066	-0.0029	12	6	7	11	6	6
3538.8562	3538.8557	0.0005	8	4	4	7	3	5
3540.5943	3540.5947	-0.0004	12	5	8	11	5	7
3541.1157	3541.1164	-0.0007	12	6	6	11	6	5
3603.4065	3603.4125	-0.0060	13	2	11	12	3	10
3608.7862	3608.7867	-0.0005	12	5	7	11	5	6
3625.5270	3625.5264	0.0006	13	3	11	12	3	10
3631.6745	3631.6729	0.0016	8	3	5	7	2	6
3634.7668	3634.7667	0.0001	12	3	9	11	3	8
3646.8757	3646.8763	-0.0006	13	2	11	12	2	10
3684.3590	3684.3549	0.0041	10	4	7	9	3	6
3696.1530	3696.1511	0.0019	14	1	13	13	2	12
3696.6262	3696.6267	-0.0005	14	2	13	13	2	12
3697.2489	3697.2493	-0.0004	14	1	13	13	1	12
3697.7281	3697.7250	0.0031	14	2	13	13	1	12
3701.0715	3701.0657	0.0058	16	12	5	16	11	6
3702.9068	3702.9071	-0.0003	12	4	8	11	4	7
3708.7836	3708.7773	0.0063	15	12	4	15	11	5
3763.7854	3763.7883	-0.0029	15	1	15	14	1	14

3765.2981	3765.2987	-0.0006	8	5	4	7	4	3
3767.3560	3767.3545	0.0015	13	4	10	12	4	9
3783.3232	3783.3235	-0.0003	8	5	3	7	4	4
3785.6766	3785.6800	-0.0034	13	10	3	12	10	2
3793.0723	3793.0806	-0.0083	13	9	4	12	9	3
3818.9240	3818.9246	-0.0006	13	7	7	12	7	6
3820.4790	3820.4808	-0.0018	13	7	6	12	7	5
3822.0466	3822.0465	0.0001	11	4	8	10	3	7
3835.3909	3835.3926	-0.0017	13	5	9	12	5	8
3837.2777	3837.2809	-0.0032	13	6	8	12	6	7
3856.6151	3856.6138	0.0013	13	6	7	12	6	6
3862.9290	3862.9251	0.0039	14	2	12	13	3	11
3873.7479	3873.7435	0.0044	14	3	12	13	3	11
3880.7378	3880.7366	0.0012	13	3	10	12	3	9
3885.0377	3885.0390	-0.0013	14	2	12	13	2	11
3895.8585	3895.8575	0.0010	14	3	12	13	2	11
3911.0633	3911.0606	0.0027	14	3	11	13	4	10
3922.8001	3922.8007	-0.0006	9	4	5	8	3	6
3941.4127	3941.4074	0.0053	15	1	14	14	2	13
3941.6119	3941.6101	0.0018	15	2	14	14	2	13
3941.8870	3941.8830	0.0040	15	1	14	14	1	13
3942.0909	3942.0857	0.0052	15	2	14	14	1	13
3950.2012	3950.1989	0.0023	12	4	9	11	3	8
3951.2934	3951.3009	-0.0075	13	5	8	12	5	7
4008.7891	4008.7896	-0.0005	16	0	16	15	0	15
4015.6912	4015.6900	0.0012	13	4	9	12	4	8
4030.8680	4030.8662	0.0018	14	4	11	13	4	10
4102.9629	4102.9625	0.0004	8	6	3	7	5	2
4103.6412	4103.6336	0.0076	8	6	2	7	5	3
4113.1113	4113.1107	0.0006	14	3	11	13	3	10
4115.0778	4115.0749	0.0029	15	2	13	14	3	12
4120.2072	4120.2068	0.0004	15	3	13	14	3	12
4123.5264	4123.5312	-0.0048	14	7	8	13	7	7
4124.1966	4124.1961	0.0005	14	5	10	13	5	9
4125.8947	4125.8934	0.0013	15	2	13	14	2	12
4127.7251	4127.7239	0.0012	14	7	7	13	7	6

4138.4707	4138.4722	-0.0015	7	7	0	6	6	1
4141.3567	4141.3556	0.0011	14	6	9	13	6	8
4253.7894	4253.7886	0.0008	17	0	17	16	0	16
4288.0916	4288.0908	0.0008	15	4	12	14	4	11
4296.3914	4296.3947	-0.0033	14	5	9	13	5	8
4307.1379	4307.1355	0.0024	14	4	10	13	4	9
4363.3253	4363.3293	-0.0040	16	2	14	15	3	13
4365.6993	4365.7035	-0.0042	16	3	14	15	3	13
4368.4683	4368.4611	0.0072	16	2	14	15	2	13
4370.8443	4370.8353	0.0090	16	3	14	15	2	13
4405.6431	4405.6424	0.0007	15	5	11	14	5	10
4408.7922	4408.7934	-0.0012	15	8	7	14	8	6
4428.5072	4428.5017	0.0055	8	7	2	7	6	1
4428.5072	4428.5131	-0.0059	8	7	1	7	6	2
4429.8328	4429.8324	0.0004	15	7	9	14	7	8
4431.4656	4431.4688	-0.0032	17	2	16	16	2	15
4431.5220	4431.5186	0.0034	17	1	16	16	1	15
4440.0298	4440.0285	0.0013	15	7	8	14	7	7
4443.3292	4443.3240	0.0052	15	6	10	14	6	9
4498.7883	4498.7891	-0.0008	18	1	18	17	1	17
4540.4088	4540.4133	-0.0045	16	4	13	15	4	12
4741.1339	4741.1329	0.0010	16	6	11	15	6	10
4743.7898	4743.7912	-0.0014	19	1	19	18	1	18
4752.8402	4752.8444	-0.0042	8	8	0	7	7	1
4789.2858	4789.2886	-0.0028	17	4	14	16	4	13
4988.7958	4988.7939	0.0019	20	0	20	19	0	19
5042.9349	5042.9344	0.0005	9	8	1	8	7	2
5233.7929	5233.7968	-0.0039	21	0	21	20	0	20
5246.9990	5247.0002	-0.0012	17	5	12	16	5	11
5367.2107	5367.2141	-0.0034	9	9	0	8	8	1
5981.5810	5981.5822	-0.0012	10	10	0	9	9	1

4.2 Rotor fragment (r-B)

Table 2: Observed and calculated rotational transitions (MHz) for the rotor fragment. The strong a-type character of r-B allowed observation of rotational transitions up to about 7 GHz.

Observed	Calculated	Obs-Calc	J′	K_a′	K_c′	J″	K_a″	K_c″
2416.4063	2416.4064	-0.0001	3	1	3	2	1	2
2557.9368	2557.9348	0.0020	3	0	3	2	0	2
2754.2838	2754.2811	0.0027	3	1	2	2	1	1
3213.1308	3213.1313	-0.0005	4	1	4	3	1	3
3374.3878	3374.3838	0.0040	4	0	4	3	0	3
3661.8902	3661.8895	0.0007	4	1	3	3	1	2
4003.4020	4003.4024	-0.0004	5	1	5	4	1	4
4164.9709	4164.9781	-0.0072	5	0	5	4	0	4
4299.2496	4299.2444	0.0052	5	2	4	4	2	3
4451.6250	4451.6328	-0.0078	5	2	3	4	2	2
4786.7771	4786.7781	-0.0010	6	1	6	5	1	5
4931.8369	4931.8396	-0.0027	6	0	6	5	0	5
5144.1726	5144.1695	0.0031	6	2	5	5	2	4
5392.7993	5392.7981	0.0012	6	2	4	5	2	3
5442.7894	5442.7886	0.0008	6	1	5	5	1	4
5563.3778	5563.3812	-0.0034	7	1	7	6	1	6
5681.8166	5681.8158	0.0008	7	0	7	6	0	6
5981.1978	5981.1981	-0.0003	7	2	6	6	2	5
6307.5595	6307.5630	-0.0035	7	1	6	6	1	5
6333.8106	6333.8023	0.0083	8	1	8	7	1	7
6342.1723	6342.1731	-0.0008	7	2	5	6	2	4
6423.1986	6423.2025	-0.0039	8	0	8	7	0	7
7148.8597	7148.8563	0.0034	8	1	7	7	1	6

References

[1] D. Schmitz, V. Alvin Shubert, T. Betz, M. Schnell, *J. Mol. Spec.* **2012**, *280*, 77–84.

[2] J. Vicario, A. Meetsma, B. L. Feringa, *Chem. Commun.* **2005**, 5910–5912.

[3] C. Pérez, S. Lobsiger, N. A. Seifert, D. P. Zaleski, B. Temelso, G. C. Shields, Z. Kisiel, B. H. Pate, *Chem. Phys. Lett.* **2013**, *571*, 1 – 15.

[4] S. Grimme, J. Antony, S. Ehrlich, H. Krieg, *J. Chem. Phys.* **2010**, *132*, 154104.

[5] S. Grimme, S. Ehrlich, L. Goerigk, *J. Comp. Chem.* **2011**, *32*, 1456–1465.

[6] Y. Zhao, D. G. Truhlar, *Theor. Chem. Acc.* **2008**, *120*, 215–241.

[7] M. Frisch, et al., *Gaussian 09* **2009**.
